# Circulating intercellular adhesion molecules in blood and bronchoalveolar lavage in Behçet's disease

**DOI:** 10.1155/S0962935195000573

**Published:** 1995-09

**Authors:** A. Hamzaoui, K. Hamzaoui, A. Chabbou, K. Ayed

**Affiliations:** 1 Department of Respiratory Diseases Faculté de Médecine de Tunis 9 rue Z. Essafi Tunis 1007 Tunisia; 2 Department of Histology Faculté de Médecine de Tunis 9 rue Z. Essafi Tunis 1007 Tunisia; 3 Department of Respiratory Diseases Faculté de Médecine de Tunis 9 rue Z. Essafi Tunis 1007 Tunisia; 4 Department of Immunology Faculté de Médecine de Tunis 9 rue Z. Essafi Tunis 1007 Tunisia

## Abstract

The aim of this study was to evaluate circulating intercellular adhesion molecule-1 (cICAM-1) in serum and in bronchoalveolar lavage (BAL), as a marker for the inflammatory process in patients with active Behçet's disease (BD). Circulating ICAM-1 was tested by an enzyme linked immuno-sorbent assay in serum and in BAL of patients with BD. These values were compared to those of patients with tuberculosis and to healthy controls. Increased levels of circulating ICAM-1 were found in serum from patients with active BD compared to healthy controls (p < 0.01). Similar levels of serum cICAM-1 were found in BD and tuberculosis. Additionally, both BD and tuberculosis patients exhibited high levels of cICAM-1 in BAL fluid, suggesting that this increase may be a result of the immune system activation in inflammatory sites. Circulating ICAM-1 seemed to have a good discriminative power in identifying active BD, being elevated in all active stages (p < 0.01) compared to remission BD stage. No differences were found in active BD patients depending upon the clinical manifestations. These results suggest that cICAM-1 may be involved in leucocyte adhesion and migration into the vessel wall of the lung. Circulating forms are derived from molecules expressed on the surface of activated cells, as a result of an inflammatory process.


Mediators of Inflammation 4, 355–358 (1995)

### Circulating intercellular adhesion molecules in blood and bronchoalveolar lavage in Behçet's disease

A. Hamzaoui,[1] K. Hamzaoui,[2,CA] A. Chabbou[3] and K. Ayed[4]

[1]Department of Respiratory Diseases, [2]Department of Histology, [3]Department of Respiratory Diseases and [4]Department of Immunology, Faculté de Médecine de Tunis, 9 rue Z. Essafi, 1007 Tunis, Tunisia

[CA]Corresponding Author

**The aim of this study was to evaluate circulating intercellular adhesion molecule-1 (cICAM-1) in serum and in bronchoalveolar lavage (BAL), as a marker for the inflammatory process in patients with active Behçet's disease (BD). Circulating ICAM-1 was tested by an enzyme linked immunosorbent assay in serum and in BAL of patients with BD. These values were compared to those of patients with tuberculosis and to healthy controls. Increased levels of circulating ICAM-1 were found in serum from patients with active BD compared to healthy controls ($p < 0.01$). Similar levels of serum cICAM-1 were found in BD and tuberculosis. Additionally, both BD and tuberculosis patients exhibited high levels of cICAM-1 in BAL fluid, suggesting that this increase may be a result of the immune system activation in inflammatory sites. Circulating ICAM-1 seemed to have a good discriminative power in identifying active BD, being elevated in all active stages ($p < 0.01$) compared to remission BD stage. No differences were found in active BD patients depending upon the clinical manifestations. These results suggest that cICAM-1 may be involved in leucocyte adhesion and migration into the vessel wall of the lung. Circulating forms are derived from molecules expressed on the surface of activated cells, as a result of an inflammatory process.**

**Key words:** Adhesion molecules, Behçet's disease, Bronchoalveolar lavage.

### Introduction

Behçet's disease (BD) is now recognized as a systemic disease with the vascular complications of aneurysm formation and venous thrombosis.[1,2] The underlying pathological process is a multifocal vasculitis involving veins, capillaries and arteries.[3,4] Involvement of the pulmonary vascular tree in BD has been extensively studied by Erkan and Cavdar[4] who evaluated its frequency at 5–10%. The pathological process affecting arteries in BD was considered a result of immune complex deposition in small vessels leading to complement fixation and polymorphonuclear cell activation, resulting in degeneration and occlusion of vasa vasorum. More complex cellular pathways are actually considered as the infiltrating cells invading inflammatory sites are mainly $CD4^+$ lymphocytes, macrophages and B cells, present in the vascular adventia and media.[5,6] These inflammatory cells together with endothelial cells produce cytokines,[6] some of which, such as interleukin-1 (IL-1), tumour necrosis factor (TNF-$\alpha$), interleukin-8 (IL-8) and interferon-gamma (IFN-$\gamma$), act as leukocyte chemotactic factors, enhance adhesion molecule expression and mediate endothelial cell damage, through increased production of oxygen free radicals.[7,8] Our previous studies showed that integrins were increased in sera, in cerebrospinal fluid and in bronchoalveolar lavage cells from BD patients.[9,10] Among several adhesion molecules that mediate adhesive interactions, intercellular adhesion molecule-1 (ICAM-1), and its ligand are known to play a pivotal role in both the migration of activated lymphocytes across endothelium and basement membranes and their adherence to target tissues where putative antigens are located. In addition to membrane-bound ICAM-1, a circulating, functionally active form of this molecule has been described, and is denoted cICAM-1.

In the present study we searched for elevated levels of cICAM-1 in sera and in bronchoalveolar lavage (BAL) from patients with active BD. Two groups were studied, tuberculous patients and healthy controls.

© 1995 Rapid Science Publishers

### Materials and Methods

*Patients:* Forty patients with BD were characterized according to the criteria of the International Study Group of Behçet's Disease.[11] Twenty-eight were in active stage, and had recurrent oral and genital ulcerations and uveitis associated with one or more other clinical manifestations as shown in Table 1. These patients were not treated. Eleven patients with active BD were suspected of having pulmonary manifestations and underwent a bronchofibroscopy with bronchoalveolar lavage. Macroscopic lung involvement was confirmed in seven cases (chronic cough associated to interstitial shadows on the chest roentgenogram or pulmonary aneurysms). As control subjects, ten patients (male, mean age 45 years, range 25–49 years) with no evidence of interstitial lung disease were studied. They were undergoing routine bronchoscopy for suspected bronchial carcinoma. In all of them, the lung lavage was roentgenographically normal, and BAL cytology showed a normal differential count. Another seven patients (male, mean age 50 years, range 37–56 years) with confirmed pulmonary tuberculosis were also studied. Informed consent was obtained from all subjects for the study.

*Immunoassays:* Circulating ICAM-1 levels were measured by a commercially available ELISA system (Bender & Co., Vienna). This kit uses a horseradish peroxidase conjugated monoclonal anti-human cICAM-1 antibody. The company's suggested procedures were followed without modification, and the plates were read at a wavelength of 450 nm in an ELISA reader. Circulating ICAM-1 concentrations were determined by comparing the mean absorption of duplicate samples with that of a standard curve. Forty healthy donors (aged 45 ± 9 years) served as controls to establish normal values of cICAM-1.

*Peripheral blood samples:* Peripheral venous blood was taken from all patients and from all control subjects. Serum was separated from clotted whole blood after centrifugation at 400 × **g** for 10 min at room temperature and stored at −70°C before measuring circulating adhesion molecules.

*Bronchoalveolar lavage:* The chosen lobe was lavaged with 50 ml aliquots of sterile 0.9% sodium chloride, as reported recently.[12] The lavage fluid was gently aspirated after each aliquot and collected into a sterile, siliconized glass bottle and maintained at 4°C. The lavage

Table 1. Clinical manifestations of active Behçet's disease

| Patient | Age | Sex | Oral | Genital | Ocular | Skin lesions[a] | CNS[b] | Pulmonary[c] |
|---|---|---|---|---|---|---|---|---|
| 1 | 32 | M | + | + | + | – | – | – |
| 2 | 45 | M | + | + | + | – | – | + |
| 3 | 25 | M | + | + | + | – | – | + |
| 4 | 36 | M | + | + | + | + | – | – |
| 5 | 27 | M | + | + | + | + | – | + |
| 6 | 48 | M | + | + | + | + | + | – |
| 7 | 29 | M | + | + | + | + | – | + |
| 8 | 43 | M | + | + | + | + | – | + |
| 9 | 33 | M | + | + | + | – | – | – |
| 10 | 43 | M | + | + | + | + | + | – |
| 11 | 39 | M | + | + | – | + | – | + |
| 12 | 38 | F | + | + | + | – | – | + |
| 13 | 42 | M | + | + | + | – | – | – |
| 14 | 52 | M | + | + | – | – | – | + |
| 15 | 33 | M | + | + | + | – | – | – |
| 16 | 35 | F | + | + | – | – | + | + |
| 17 | 36 | M | + | + | + | + | + | + |
| 18 | 27 | M | + | + | + | + | – | – |
| 19 | 48 | M | + | + | – | + | – | – |
| 20 | 37 | M | + | + | – | + | – | – |
| 21 | 46 | M | + | – | + | + | + | – |
| 22 | 38 | M | + | – | + | + | – | – |
| 23 | 25 | M | + | + | + | – | + | + |
| 24 | 34 | M | + | + | + | – | + | – |
| 25 | 29 | M | + | + | – | + | – | – |
| 26 | 32 | M | + | + | – | – | – | – |
| 27 | 27 | M | + | + | – | – | – | – |
| 28 | 36 | M | + | + | – | – | – | – |

[a]Erythema nodosum, cutaneous, vasculitis, pyoderma or pustules.
[b]Meningoencephalocele, seizures, cranial nerve palsy, quadriparesis.
[c]Pulmonary interstitial shadows (aneurysm on the right main branch or on the upper lobe pulmonary artery).

fluid was filtered through coarse gauze and centrifuged at 350 × ***g*** at 4°C. The supernatants were concentrated by evaporation and reconstituted in 1 ml normal saline and studied by ELISA for cICAM-1.

*Statistical analysis:* All results are expressed as mean ± standard deviation (S.D.). Only $p < 0.01$ degree of significance was accepted. The Mann–Whitney *U*-test was used to compare the levels of circulating adhesion molecules between the different groups.

### Results

*Serum levels of cICAM-1:* Circulating ICAM-1 was detected in all normal healthy controls (350 ± 24 ng/ml) (Fig. 1). Patients with active tuberculosis exhibited high levels of cICAM-1 molecule (570 ± 96 ng/ml), when compared to healthy controls (Fig. 1). Patients with BD showed high levels of cICAM-1 (584 ± 47 U/ml), when compared to healthy controls ($p < 0.001$) (Fig. 1 and Table 2). No differences were observed in serum cICAM-1 between BD patients and tuberculous patients.

Patients with BD were studied according to their clinical stage. Patients with active BD had high levels of circulating cICAM-1 (694 ± 37 ng/ml), when compared to BD patients in remission stage (387 ± 69 ng/ml) (Table 2). Patients with active BD were compared for circulating adhesion molecules, according to their clinical manifestations. No differences were found between patients with pulmonary or with extrapulmonary manifestations (Table 2).

Table 2. Circulating ICAM-1 in serum from patients with BD

| Diagnosis | No. | cICAM-1 (ng/ml) |
|---|---|---|
| BD patients | 40 | 582 ± 47* |
| BD in active stage | 28 | 694 ± 37* |
| BD in remission stage | 12 | 387 ± 69 |
| BD with pulmonary manifestations | 10 | 652 ± 39* |
| BD with CNS involvement | 7 | 714 ± 67* |
| BD with skin lesions | 14 | 650 ± 35* |
| BD with ocular manifestations | 19 | 590 ± 93* |

cICAM-1 was increased in active BD (*$p < 0.01$), compared to BD in remission stage. No differences were found between patients with pulmonary manifestations and extrapulmonary involvements. Values are expressed as mean ± S.D.

*BAL fluid level of cICAM-1:* BAL fluid from ten BD patients, seven tuberculous patients and ten healthy controls was studied for circulating adhesion molecules (Fig. 2). BAL fluid from healthy controls showed limit levels of cICAM-1 (105 ± 28 ng/ml). BD patients with pulmonary manifestations exhibited high levels of cICAM-1 (635 ± 47 ng/ml). Levels in BAL fluid from patients with tuberculosis (734 ± 96 ng/ml) were increased to levels similar to those in BD patients.

### Discussion

Circulating ICAM-1 levels in serum and in BAL fluid from patients with active BD and having

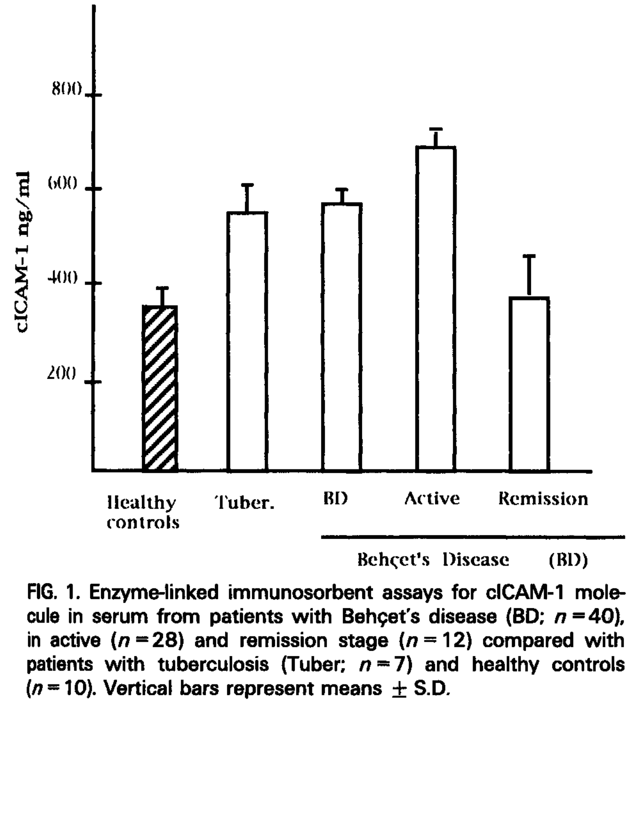

FIG. 1. Enzyme-linked immunosorbent assays for cICAM-1 molecule in serum from patients with Behçet's disease (BD; $n = 40$), in active ($n = 28$) and remission stage ($n = 12$) compared with patients with tuberculosis (Tuber; $n = 7$) and healthy controls ($n = 10$). Vertical bars represent means ± S.D.

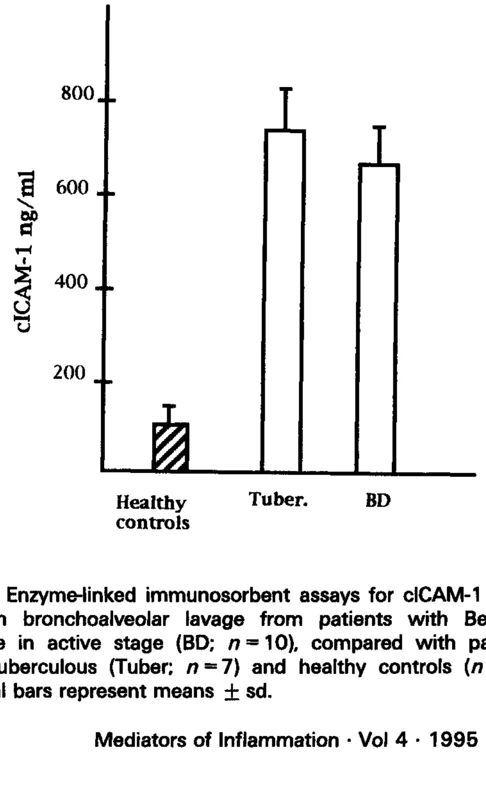

FIG. 2. Enzyme-linked immunosorbent assays for cICAM-1 molecule in bronchoalveolar lavage from patients with Behçet's disease in active stage (BD; $n = 10$), compared with patients with tuberculous (Tuber; $n = 7$) and healthy controls ($n = 10$). Vertical bars represent means ± sd.

pulmonary manifestations were increased at the same level as in tuberculous patients, compared to healthy controls. The pathological importance of circulating adhesion molecules has not yet been fully delineated. The release of ICAM-1 from the surface may be a mechanism to control cell adhesion. Circulating ICAM-1 may be a byproduct of alteration in cell adhesion molecule expression and cell adhesion, characteristic of inflammation. BD in the active stage was characterized by an increase of cICAM-1 compared to BD in the remission phase. The elevated serum concentration of ICAM-1 in active BD suggests that it may be a useful marker of disease activity. Thus, circulating ICAM-1 was raised in all active BD patients, independently of the lesion sites. This increase was specific to the activated stage, reflecting immune system activation.

The most important point was the increased cICAM-1 in BAL fluid from BD patients. A similar increase was previously reported in pulmonary tuberculosis[13] and idiopathic pulmonary fibrosis,[13] that are characterized by extensive inflammation. In BD, lymphocytes are responsible for the recruitment of inflammatory cells to the lung,[14] by releasing a wide array of cytokines. Patients with active BD are characterized by an increase of INF-γ,[15] TNF-α,[8,16] IL-8,[8] and IL-6[8,12] production. These cytokines can up-regulate adhesion molecule expression on some endothelial cells.[17] The presence of circulating adhesion molecules in BAL from BD patients provides a possible mechanism by which these inflammatory leukocytes are attracted to the lesion site, leading to development of the disease. The main features of lung involvement in BD consist of vasculitis forming multifocal aneurysm and thrombosis of the pulmonary artery.[18–20] Histological examination of the aneurysm wall reveals an inflammatory exudate containing lymphocytes and polymorphonuclear leukocytes invading the adventia of the artery.[17] ICAM-1, the LFA-1 receptor of which is expressed on all leukocytes,[21] probably mediates the adhesion of neutrophils, monocytes and lymphocytes to vascular endothelium. LFA-1 was found to be increased in BAL lymphocytes from active BD patients,[9] facilitating their transendothelial migration. Atherosclerotic aortic aneurysms and plaques[21–23] are also associated with elevated cICAM-1. The detection of cICAM-1 may serve as a useful diagnostic tool in diseases where leukocyte endothelial cell interactions play an important role.

### References

1. Barlett ST, McCarthy JW, Palmer AS, Flinn RW, Bergman JJ, Yao ST. Multiple aneurysms in Behçet's disease. *Arch Surg* 1988; **123:** 1004–1007.
2. O'Duffy JD. Pulmonary involvement in Behçet's disease. *Eur Respir J* 1993; **6:** 936–937.
3. Gamble CN, Wiesner KB, Shapiro RE, Boyer WJ. The immune complex pathogenesis of glomerulonephritis and pulmonary vasculitis in Behçet's disease. *Am J Med* 1979; **66:** 1031–1039.
4. Erkan F, Cavdar T. Pulmonary vasculitis in Behçet's disease. *Am Rev Respir Dis* 1992; **146:** 232–239.
5. Koch AE, Haines GK, Rizzo RJ, Radosevich JA, Pope RM, Robinson PG, Pearce WH. Human abdominal aortic aneurysms: immunophenotypic analysis suggesting an immune-mediated response. *Am J Pathol* 1990; **137:** 1199–1213.
6. Ross R. Mechanisms of atherosclerosis—a review. *Adv Nephrol* 1990; **19:** 79–86.
7. Niwa Y, Miyake S, Sakan ET. Autoaxiclotieve damage in Behçet's disease: endothelial cell damage following elevated oxygen radicals generated by stimulated neutrophils. *Clin Exp Immunol* 1982; **49:** 247–255.
8. Mege JL, Dilsen N, Sanguedolce V, *et al.* Overproduction of monocyte derived tumor necrosis factor α, interleukin (IL) 6, IL-8 and increased neutrophil superoxide generation in Behçet's disease. A comparative study with familial Mediterranean fever and healthy subjects. *J Rheumatol* 1993; **20:** 1544–1549.
9. Hamzaoui K, Hamzaoui A, Hentati F, *et al.* Phenotype and functional profile of T cells expressing γδ receptor from patients with active Behçet's disease. *J Rheumatol* 1994; **21:** 2301–2306.
10. Hamzaoui K, Hentati F, Hamzaoui A, Kahan A, Ben Hamida M, Chabbou A, Ayed Kh. CD11/CD18 bearing lymphocytes in cerebrospinal fluid from patients with active Behçet's disease. *Clin Exp Rheumatol* 1994; **12:** 575–576.
11. International Study Group (ISG) for Behçet's disease. Criteria for diagnosis of Behçet's disease. *Lancet* 1990; **335:** 1078–1080.
12. Hamzaoui K, Hamzaoui A, Kahan A, Hamza M, Chabbou A, Ayed Kh. Interleukin-6 in peripheral blood and inflammatory sites in Behçet's disease. *Mediators of Inflammation* 1992; **1:** 181–185.
13. Lai CKW, Wong KC, Chan HSC, Chung H, Hasard DO, Lai KN. Circulating adhesion molecules in tuberculosis. *Clin Exp Immunol* 1993; **94:** 522–526.
14. Shijubo N, Imai K, Aoki S. Circulating intercellular adhesion molecule-1 (ICAM-1) antigen in sera of patients with idiopathic pulmonary fibrosis. *Clin Exp Immunol* 1992; **89:** 58–62.
15. Hamzaoui K, Ayed Kh, Slim A, Hamza M, Touraine JL. Natural killer activity, interferon-gamma and antibodies to herpes viruses in patients with Behçet's disease. *Clin Exp Immunol* 1990; **79:** 28–34.
16. Hamzaoui K, Hamza M, Ayed Kh. Production of TNF-α and IL-1 in active Behçet's disease. *J Rheumatol* 1990; **17:** 1428–1429.
17. Detmar M, Tenorio S, Hattmannsperger U, Ruszezak Z, Orfanos CE. Cytokine regulation of proliferation and ICAM-1 expression of human dermal microvascular endothelial cells *in vitro*. *J Clin Invest Dermatol* 1992; **98:** 147–153.
18. Leavitt RY, Fauci AS. Pulmonary vasculitis. *Am Rev Respir Dis* 1986; **134:** 149–166.
19. Bouacha H, Azzabi S, Hamzaoui A, Kanzari A, Daoues A, Maalej M. Aneurysme de l'artère pulmonaire et infarctions pulmonaires multiples inaugurant la maladie de Behçet. *Ann Med Interne* 1991; **142:** 544–546.
20. Raz I, Elimelech O, Chajek-Shaul T. Pulmonary manifestations in Behçet's syndrome. *Chest* 1989; **95:** 585–589.
21. Figdor CG, van Kooyk Y, Keizer GD. On the mode of action of LFA-1. *Immunol Today* 1990; **118;** 277–280.
22. van der Wall AC, Das PK, Tigges AJ, Becker AE. Adhesion molecules on the endothelium and mononuclear cells in human atherosclerotic lesions. *Am J pathol* 1992; **141:** 1427–1433.
23. Szekanecz Z, Shah MR, Pearce WH, Koch AE. Intercellular adhesion molecule-1 (ICAM-1) and soluble ICAM-1 (cICAM-1) production by cytokine-activated human aortic endothelial cells: a possible role for ICAM-1 and cICAM-1 in atherosclerotic aortic aneurysms. *Clin Exp. Immunol* 1994; **98:** 337–343.

ACKNOWLEDGEMENT. This study was financially supported by the 'Secrétariat d'Etat à la Recherche Scientifique et Technique' of Tunisia

**Received 14 June 1995; accepted 22 June 1995**
